# Adverse Events of Auricular Therapy: A Systematic Review

**DOI:** 10.1155/2014/506758

**Published:** 2014-11-10

**Authors:** Jing-Yu Tan, Alexander Molassiotis, Tao Wang, Lorna K. P. Suen

**Affiliations:** ^1^School of Nursing, The Hong Kong Polytechnic University, Hung Hom, Kowloon, Hong Kong; ^2^The Second Affiliated People's Hospital, Fujian University of Traditional Chinese Medicine, No. 13 Hudong Road, Gulou District, Fuzhou 350003, China

## Abstract

The aim of this study was to systematically evaluate the literature on adverse events associated with auricular therapy (AT). Case reports, case series, surveys, and all types of clinical trials reporting adverse events of AT were included. Relevant articles were mainly retrieved from 13 electronic databases and seven Chinese journals on complementary medicine. AT-related adverse events were reported in 32 randomized controlled trials, five uncontrolled clinical trials, four case reports, and two controlled clinical trials. For auricular acupuncture, the most frequently reported adverse events were tenderness or pain at insertion, dizziness, local discomfort, minor bleeding and nausea, and so forth. For auricular acupressure, local skin irritation and discomfort, mild tenderness or pain, and dizziness were commonly reported. Skin irritation, local discomfort, and pain were detected in auricular electroacupuncture, and minor infection was identified in auricular bloodletting therapy. Most of these events were transient, mild, and tolerable, and no serious adverse events were identified. Our findings provide preliminary evidence that AT is a relatively safe approach. Considering the patient's safety, prospective or retrospective surveys are needed in future research to gather practitioner-reported and patient-reported adverse events on AT, and the quality of adverse events reporting in future AT trials should be improved.

## 1. Introduction

Being one of the most popular complementary therapeutic approaches, auricular therapy (AT) is defined as “a health care modality whereby the external surface of the ear, or auricle, is stimulated to alleviate pathological conditions in other parts of the body” [1]. The earliest record of AT can trace back to 500 to 300 B.C. in ancient China, where the* Yellow Emperor's Inner Canon* (*Huangdi Neijing*) described that the ear is not isolated but intimately connected with the five viscera and the six bowels [2]. The modern system of AT was developed by the French neurologist Paul Nogier in the late 1950s, and it is recognized that the outer ear has a somatotopic arrangement with an inverted fetus pattern and each internal organ is corresponding to a sensitive point located in the auricle [3]. AT could produce a therapeutic effect for treating various types of disorders by stimulating the particular acupoint which corresponds to the targeted part of the body or organ [4]. Various modalities are adopted in AT practice including auricular acupuncture, acupressure, moxibustion, injection, and auricular bloodletting therapy.

The WHO recognizes AT as a microacupuncture system that can produce a positive impact on regulating the whole body function [5], and its therapeutic effect has been investigated in a wide range of health problems in both oriental and western countries. Clinical trials and systematic reviews have shown that AT can be a promising modality in relieving preoperative anxiety [6], psychosomatic disorders [7], and various types of pain [8], managing hypertension [9] and cocaine dependence [10], and controlling obesity [11]. The wide use of AT in clinical practice requires continual safety evaluation. It is said that the popularity of AT is partially attributed to its convenience and safety, and in some oriental countries, AT is usually conducted by healthcare professionals only with AT short-term training or even by unqualified practitioners without any experience in performing AT [12]. However, this kind of intervention is not entirely risk-free, where adverse events, such as chest tightness, dizziness, perichondritis, and nausea, are also reported in the literature [13]; meanwhile, when applying AT to special populations such as pregnant women and immunocompromised patients, unwanted miscarriage and infection could have occurred [13].

The ear possesses abundant capillaries which make it highly vulnerable to skin inflammations and other infections [13]. To minimize potential harms caused by AT, practitioners need to strictly follow standardized procedures of AT administration as well as fully understand the potential adverse events associated with it. It is important to assess the safety of AT in clinical practice. Unfortunately, different from other traditional therapies such as body acupuncture, moxibustion, and cupping, whose safety is well analyzed in surveys and/or systematic reviews [14–17], clinical evidence on the safety of AT has not been clearly established to date. Despite the increasing number of studies in recent years reporting harm data associated with AT, their results have not been systematically summarized. Up to now, there is no systematic review to evaluate the adverse events associated with AT. Therefore, the aim of this study was to evaluate the type and frequency of AT-related adverse events, to identify any avoidable adverse events associated with nonstandardized AT procedures, and to provide recommendations for future research and practice in this area.

## 2. Methods

A study protocol accompanied with a data extraction form was formulated and critically reviewed by two experts who were familiar with AT and systematic reviews before the initiation of the study.

### 2.1. Definition of Adverse Events

In this study, an adverse event was defined as “an undesirable experience associated with the use of a medical product in a patient” according to the US Food and Drug Administration [18]. A serious adverse event was defined when the event led to serious outcomes such as death, hospitalization, disability, and permanent damage or as being life-threatening [18].

### 2.2. Inclusion and Exclusion Criteria

Case reports, case series, prospective and retrospective surveys, and all types of clinical trials (randomized controlled trials, nonrandomized controlled clinical trials, or other uncontrolled clinical trials) reporting adverse events associated with AT in human subjects were included. Animal research, in vitro studies, and review papers were excluded. Types of AT could be auricular acupuncture, auricular electroacupuncture, auricular acupressure, auricular moxibustion, auricular injection, or auricular bloodletting therapy.

### 2.3. Data Sources and Searching Strategies

Relevant studies were retrieved from three sources: electronic database, manual search, and reference lists search for final included studies. Electronic search was conducted in 13 databases (from inception to May 8, 2014) including PubMed, EMBase, Cochrane Central Register of Controlled Trials (CENTRAL), CINAHL, PsycINFO, Allied and Complementary Medicine (AMED), Thomson Reuters Web of Science, Science Direct, Foreign Medical Journal Service (FMJS), China National Knowledge Infrastructure (CNKI), WanFang Data, Chinese Scientific Journal Database (CQVIP), and Chinese Biomedical Literature Database (CBM). No language restriction was applied for electronic search. Meanwhile, seven Chinese core journals on complementary medicine (Journal of Integrative Medicine, Chinese Acupuncture & Moxibustion, China Journal of Traditional Chinese Medicine and Pharmacy, Journal of Traditional Chinese Medicine, Chinese Journal of Basic Medicine in Traditional Chinese Medicine, Chinese Journal of Integrated Traditional and Western Medicine, Journal of Beijing University of Traditional Chinese Medicine, and Journal of Nanjing University of Traditional Chinese Medicine, issues within the latest three years) were manually retrieved for further relevant articles. Reference lists of the included papers were also checked to identify any potential eligible studies. All searches were conducted by two reviewers (Tan JY and Wang T) independently. Mesh terms, keywords, and free words such as “auriculotherapy,” “acupuncture, ear,” “adverse event^*^,” “side effect^*^,” “adverse effect^*^,” “adverse health care event,” “safe^*^,” and “risk^*^” were used in the searching strategies.  Table 3 presents three main search strategies for this review.

### 2.4. Study Selection and Data Extraction

Study characteristics and outcome data of each included article were extracted using the data extraction form, which included (1) first author, year of publication, study design, and setting; (2) participant characteristics (age, gender, sample size, diagnostic criteria, and reason for AT); (3) AT protocol (type of AT, practitioner, selected acupoints, type of AT equipment, and treatment duration); and (4) AT-related adverse events (type of adverse event, frequency, outcome, and causality). Study selection and data extraction were conducted by two reviewers independently, and disagreement was resolved through discussion.

### 2.5. Outcome Assessment

Type and frequency of AT-related adverse events were the main outcomes of this study. The likelihood of causality and quality of adverse events reporting were also judged by two reviewers and checked by two experts specialized in AT and acupuncture.

For adverse events reported in case reports or case series, the likelihood of causality was judged by* the WHO-Uppsala Monitoring Centre (UMC) System for Standardized Case Causality Assessment* [19]. The likelihood was classified into six grades including the following: (1) certain: a plausible time relationship that adverse events clearly occurred after receiving AT and disappeared after withdrawal, and these events could not be explained by other health problems or interventions; (2) probable/likely: a reasonable time relationship that the onset of symptoms was most likely related to AT and that was unlikely attributed to other health problems or interventions; (3) possible: a reasonable time relationship that the onset of symptoms was most likely related to AT but that could also be explained by other health problems or interventions, and the information on AT withdrawal was lacking or unclear; (4) unlikely: there was an improbable time relationship between AT and the adverse event; (5) conditional/unclassified: event occurred but more data were essential for a proper causality assessment; and (6) unassessable/unclassifiable: an adverse event was suggested by a report but cannot be judged due to insufficient or contradictory information [19].

For clinical trials, the quality of adverse events reporting was assessed using* the CONSORT for Harms Data Recommendations* [20, 21]. Seven items were employed for assessment [17, 20, 21]: (1) report of data on harms in the title or abstract; (2) report of AT-related harms in the introduction section; (3) prespecification of potential adverse events of AT (clinical and/or laboratory); (4) specification of approach for collecting harm-related information; (5) description of plans for presenting and analyzing adverse events of AT; (6) description of participant withdrawals due to adverse events of AT; and (7) report of the particular denominators for analyses on AT-related harms. The quality of each item was judged as “adequate,” “partially adequate,” “inadequate,” or “not reported” accordingly [17]. It was rated as “adequate” if an item was properly described in detail in the article or in the study protocol; “partially adequate” was given if an item was properly described but only in a brief format; when an item failed to be properly described, the quality was judged as “inadequate”; and “not reported” meant an item was not described [17].

## 3. Results

### 3.1. Characteristics of Analyzed Studies

**Figure 1:**
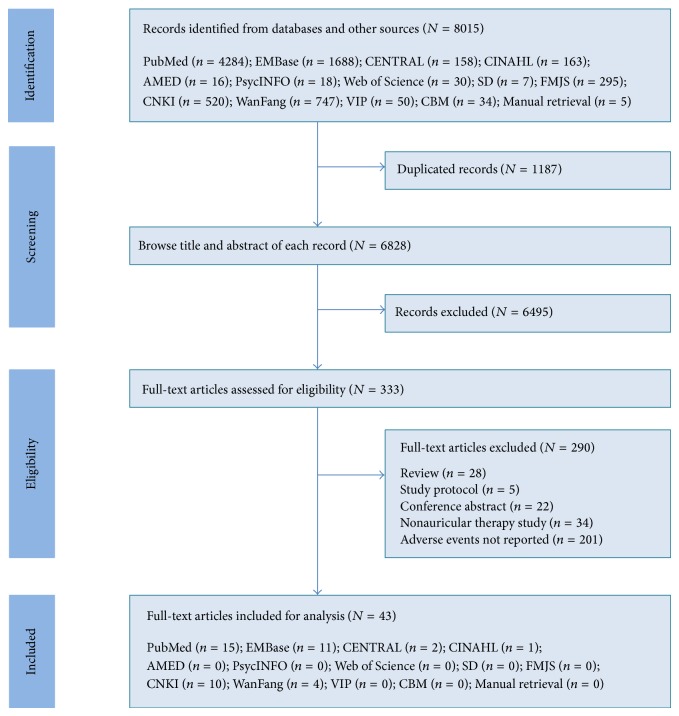
Flow chart of study selection. CENTRAL: Cochrane Central Register of Controlled Trials, CINAHL: Cumulative Index to Nursing and Allied Health Literature; AMED: Allied and Complementary Medicine, SD: Science Direct, FMJS: Foreign Medical Journal Service, CNKI: China National Knowledge Infrastructure, VIP: Chinese Scientific Journal Database, and CBM: Chinese Biomedical Literature Database.

Electronic and manual searches yielded 8015 records. After checking by reference management software, 1187 duplicated records were removed, and another 6495 were further excluded after browsing the titles and abstracts. Full text of the remaining 333 records was retrieved for eligibility assessment, and 290 articles were finally excluded because they were reviews (*n* = 28), were study protocols (*n* = 5), were conference abstracts (*n* = 22), and were non-AT interventions (*n* = 34), and the adverse events were not reported (*n* = 201). Therefore, 43 studies [22–64] were identified for final analysis. The flow chart of study selection is presented in Figure 1.

The analyzed studies included 32 randomized controlled trials, five uncontrolled clinical trials, four case reports, and two nonrandomized controlled trials, with a total of 3396 participants receiving AT treatment. Six studies were from the United States, five from Taiwan, four from Germany, three from Hong Kong, two from Australia, two from Austria, two from the United Kingdom, 15 from China, and one each from Malaysia, Canada, Sweden, and Spain. Four AT modalities were utilized including auricular acupuncture in 18 studies [26–43], auricular acupressure in 21 studies [22–25, 44–60], auricular electroacupuncture in three studies [61–63], and auricular bloodletting therapy in one study [64]. AT was applied to deal with a variety of health problems such as drug dependence, smoking cessation, pain, constipation, insomnia, and obesity.

The clinical effectiveness of AT was descriptively summarized from the included 34 controlled clinical trials (randomized or nonrandomized) [26–32, 34–42, 44–50, 52–56, 58–63], as the data synthesis was not available due to the significant clinical heterogeneity in the types of disease, AT protocols, and intervention durations among analyzed trials. Twenty-three studies reported significantly positive effect of AT for the primary and/or secondary outcomes between groups, while eight studies only detected favorable changes within the AT groups. Of the controlled clinical trials that employed auricular acupuncture, 93.8% (15/16) stated positive outcomes of AT between or within groups, whereas it was 93.3% (14/15) in studies using auricular acupressure, respectively. Two out of three trials on auricular electroacupuncture showed clinical effectiveness of AT. The majority (30 studies) described the person who administered AT including acupuncturist, TCM practitioner, physician, psychiatrist, therapist, and nurse. The selection of acupoints for treatment was based on the targeted health problem but* shenmen* was the most commonly referred acupoint which was used in 35 studies for treating various types of disorders.

### 3.2. Case Reports

Table 1 presents AT-related adverse events reported in case reports. Four cases were located and the reported adverse events were dizziness in one case [22], somnolence in two cases [23, 24], and abdominal pain in one case [25]. No serious adverse events were identified. All cases were treated with auricular acupressure using vaccaria seeds, whereas only two [22, 23] specified the practitioner who administered AT (a physician).

In Ye's report [22], a 48-year-old woman with constipation experienced dizziness five minutes after receiving auricular acupressure. The symptom gradually disappeared after removing the taped seeds. In two other reports [23, 24], two men (one was 41-year-old with lumbar muscle strain, and another was 43-year-old with dilated cardiomyopathy) reported drowsiness and somnolence at the 12th and 15th day, respectively, during the AT treatment. Their symptoms disappeared immediately after removing the taped seeds and reoccurred when seeds were taped again. Adverse events described in these three cases were assessed as probably/likely related to AT. In Ma's report [25], a 58-year-old woman with chronic diarrhea suffered from abdominal pain 30 minutes after receiving auricular acupressure, and the symptom disappeared immediately after removing the taped seeds. The author described it as a rare event caused by AT and the causality was assessed as possible.

### 3.3. Clinical Studies

#### 3.3.1. Quality of Adverse Events Reporting

Table 2 presents AT-related adverse events reported in clinical trials. For the quality of adverse events reporting, the overall results were not optimal. Twenty studies adequately or partially adequately described adverse events in the title and/or abstract (51.3%), whereas there were only nine studies appropriately describing safety issues of AT in the introduction (23.1%). AT-related adverse events were seldom prespecified (17.9%), and 16 studies properly described approaches for adverse events data collection such as investigator observation, questionnaire, or self-report (41.0%). Only six studies properly described plans for presenting and analyzing adverse events (15.4%). Twelve studies adequately or partially adequately reported whether there were any subjects that withdrew due to adverse events of AT (30.8%), and 13 studies appropriately described the denominators for analyzing adverse events (33.3%).

#### 3.3.2. Auricular Acupuncture-Related Adverse Events

Auricular acupuncture-related adverse events were reported in 18 clinical studies [26–43] with a total of 1753 participants receiving AT (Table 2). Duration of treatment varied among studies and 10 studies offered AT for more than three weeks. The most frequently reported adverse events were tenderness or pain at the needling site, dizziness, discomfort at the needling site, local bleeding, nausea, headache, and inflammation at insertion. Most of these events were transient, minor, and tolerable.

Eleven studies reported 134 cases complaining of local pain and tenderness at the needling site. AT was performed by acupuncturist in eight studies [27, 31, 32, 34–36, 38, 39], by psychiatrist and nurse in two studies [37, 41], and by physiotherapist in one study [28]. Forty-three cases withdrew due to pain but the majority could tolerate AT and completed the treatment. Two studies [32, 35] did not take any measures to decrease adverse events and symptoms gradually declined, and one study [41] stopped AT temporarily or reduced treatment frequencies to deal with local pain.

Twenty-five cases in eight studies reported minor bleeding at insertion. AT was administered by an acupuncturist in five studies [27, 35, 38, 40, 42], by psychiatrist and nurse in two studies [37, 41], and by physiotherapist in one study [28]. Bleeding often happened during inserting and/or withdrawing the needle and stopped soon without any treatment. Seven studies [27, 29, 30, 35, 36, 42, 43] reported 51 cases experiencing dizziness after receiving auricular acupuncture; one study was conducted by a TCM practitioner [43] and one by an investigator with acupuncture diploma [29], while all others were carried out by acupuncturists. One study [35] took no action for dizziness and the symptom gradually disappeared, and three studies [36, 42, 43] reported that dizziness disappeared after removing the auricular stimulation, and only one case withdrew due to dizziness.

Minor nausea was reported in five studies (22 cases). AT was provided by an acupuncturist in four studies [27, 35, 36, 42] and by a TCM practitioner in one [43]. Three studies [36, 42, 43] stated that nausea subsided after withdrawing the ear stimulation, one study [35] did not take any action, and the symptom gradually declined, whereas one study [27] failed to report the outcome. Two studies [27, 38] (AT performed by acupuncturist) reported 18 cases developing minor headache after AT, one did not report the outcome [27], and another stated that headache was resolved afterwards [38].

Two studies reported two cases experiencing minor inflammation (swelling or redness) around the needling site. AT was administered by an acupuncturist [31] and a physiotherapist [28], respectively. One study [28] stated that the subject who complained of swelling concealed a history of rheumatoid arthritis, which belonged to one of the exclusion criteria of that study. Twenty-seven cases in two studies [26, 29] reported discomfort or a strange feeling at insertion and one case withdrew. Only one study [35] reported minor infection around the needling site in one case and the condition gradually improved.

In addition, there were some other adverse events reported in a single study, including slight fever (19 cases) and dry mouth (15 cases), which focused on methadone maintenance treatment (MMT) for drug-dependent persons [27]. Transient exacerbation of vasomotor symptoms (2 cases) was found in one study which focused on the vasomotor symptoms associated with luteinizing hormone releasing hormone agonist treatment in prostate cancer patients [33]. Upper limb numbness was mentioned in one study [43] and it was resolved immediately after removing stimulation of acupoint “sympathetic.”

#### 3.3.3. Auricular Acupressure-Related Adverse Events

Auricular acupressure-related adverse events were reported in 17 clinical trials [44–60] (Table 2). A total of 1266 participants were treated with auricular acupressure (true or sham intervention) and the majority employed vaccaria seeds and/or magnetic pellets performing acupressure. More than half of the studies provided AT for no less than two weeks. The commonly reported adverse events were local skin irritation, discomfort, tenderness or pain at the taped site, and dizziness, and most of them were also mild, short-term, and well tolerated.

Thirteen studies [45, 47–50, 53–60] reported 63 cases suffering from local skin irritation with itchiness, allergy, or redness after receiving auricular acupressure. Only half of the studies specified the professional conducting AT, which include acupuncturist in two studies [53, 60], TCM practitioner in two studies [50, 58], therapist in one study [48], and nurse in the other one [54]. Skin irritation was mostly attributed to the adhesive tapes. However, there was one study [57] reporting several subjects who were allergic to magnetic pellets. Seven cases withdrew due to skin irritation, and five cases changed adhesive tape to desensitization material and treatment continued, and those subjects who were allergic to magnetic pellets switched to vaccaria seeds and symptoms disappeared. Itchiness spontaneously subsided in 25 cases, and three cases recovered after treatment, while others tolerated well these symptoms and continued to complete treatment.

Three studies [51–53] reported tenderness or pain at the taped site (16 cases), and only two [51, 53] specified the AT practitioner (acupuncturist). No subjects withdrew, but one study [52] reported five cases experiencing obvious ear pain when receiving AT for the first time, and symptoms were relieved after reducing the pressing frequency and intensity of the taped seeds.

Two studies [46, 53] reported mild to moderate discomfort at the taped site (35 cases). Apart from one case that withdrew, others tolerated well the discomfort. Another two articles [45, 46] reported three cases experiencing minor dizziness during AT treatment and one subject withdrew. In one study [54] in which AT was administered by a nurse (the author did not declare whether the nurse had received any training in AT), ear skin breakdown was recorded in one subject, and the skin recovered two days later after using entoiodine. One study [44] reported pressure ulcers in the pinna in 18 subjects after receiving auricular acupressure and all ulcers healed within 10 days after removing the tapes.

#### 3.3.4. Auricular Electroacupuncture-Related Adverse Events

Three articles [61–63] reported adverse events of auricular electroacupuncture (Table 2), which included 203 participants treated with true or sham AT. Treatment duration ranged from five to six weeks. Two studies described AT practitioners including a doctor with acupuncture experience in one [63] and nurse in another [62]. Reported adverse events included discomfort and pain at insertion and local skin irritation. All reactions were mild and tolerable.

Two studies [62, 63] mentioned two cases complaining of discomfort at the needling site but the outcome was not reported. One study [61] reported mild ear skin irritation in eight cases, and the author explained it was induced by the adhesive patch of the P-stim or placebo device. Skin irritations were resolved immediately after AT. One study [63] found one case experiencing pain at the needling site but no outcome was reported.

#### 3.3.5. Auricular Bloodletting Therapy-Related Adverse Events

There was only one study [64] using auricular bloodletting therapy and 170 subjects were included. Two cases reported minor infection at the needling site but the outcome was not described.

## 4. Discussion

Our findings provide preliminary evidence that AT is a relatively safe approach in routine practice. The most frequently reported adverse events were mainly confined to short-term, mild, and tolerable reactions such as local discomfort, transient tenderness and pain, local skin irritation, minor bleeding, and dizziness. Some of them are potentially avoidable, and no serious adverse events were detected. However, the clinical practice of AT still needs caution because some adverse events like dizziness, somnolence, and infection could also result in serious negative outcomes. Meanwhile, the quality of adverse events reporting should be improved in future research and related guidelines such as* the Guidelines for Case Reports of Adverse Events Related to Acupuncture* [65], and* the CONSORT for Harms Data Recommendations* [20] should be followed.

Complementary therapeutic approaches such as body acupuncture, moxibustion, cupping, and AT have been widely used in dealing with a variety of disorders. Apart from the increasing emphasis on their therapeutic effects, safety of these interventions also received wide attention. The safety of body acupuncture has been well summarized in the literature and a number of serious adverse events have been identified including pneumothorax, hepatitis, staphylococcus infection, and central nervous system injuries [14]. Adverse events of moxibustion were also systematically analyzed and some rare but dangerous events, for instance, burns, cellulitis, ecchymoma, and hepatitis C, were reported [14, 16]. In a recent systematic review [17], cupping-related adverse events reported in South Korea were investigated, and anemia, herpes viral infections, and skin lacerations were identified. In our findings, no serious adverse events of AT were reported and the reactions were mostly transient, mild, and tolerable. Based on our findings, the safety of AT seems superior to other traditional therapies such as body acupuncture, moxibustion, and cupping.

Various adverse events were identified in studies using auricular acupuncture, of which dizziness was one of the most commonly reported symptoms. Similar reports can also be found in body acupuncture [66–68], which viewed dizziness as mild symptoms of acupuncture-related fainting. This may be because of transient hypotension, as acupuncture stimulation through the peripheral vagus nerve reflex could dilate the peripheral blood vessels and reduce venous return [68, 69]. As a result, decreasing in the brain blood supply induces transient hypotension and causes symptoms such as dizziness and weakness [68, 69]. Dizziness was often seen in people who received AT for the first time, especially for those who felt anxious or nervous before treatment and for those with extremely weak condition or with hypoglycemia [12]. Although dizziness is generally mild and can spontaneously subside, potential risks associated with it still need caution, especially when applying AT in community or in clinical settings. Most clients there would leave the clinic immediately after finishing AT, and if dizziness occurs afterwards, accidental falls might happen. AT practitioners should pay attention to this issue, particularly for those elderly patients with osteoporosis.

As an invasive approach, auricular acupuncture may also lead to some infections. But from our findings, only one case was found with minor infection at insertion, and no serious infections such as chondritis, cellulitis, and hepatitis were detected. This finding is inconsistent with Norheim's study [70] which indicated that auricular chondritis was a commonly reported infection in patients receiving acupuncture on the ear. The infected cases came from the literature published 20–30 years ago, and the author concluded that the hygienic problem, such as insufficient needle skin disinfection, partially contributed to these infections [70]. Unqualified sterilization and disinfection for either skin or needle equipment may be one of the crucial risk factors for acupuncture-related infections at that time. In our analyzed studies, the needles used in AT were generally sterilized and disposable, and the ear skin was mostly reported well disinfected. The gradually improved awareness of strict hygienic procedures during AT could be one of the reasons for the low incidence of infection identified in our review. However, practitioners also need to prudently use this approach on clients with poor wound-healing capacity, such as patients with diabetes mellitus, extremely weak status, or immunocompromised disorders [13], as potentially local damage could result in some nonhealing wounds or even systemic infections. Considering these circumstances, other noninvasive AT modalities such as auricular acupressure could be adopted instead.

It is worth noting that there were several rare adverse events reported in one trial in which the intervention group received AT plus MMT while the control group received MMT only [27]. The author claimed that dry mouth and slight fever were AT-related adverse events. However, dry mouth is a general side effect of methadone [71], and sometimes fever is considered as one of the opioid withdrawal symptoms during the induction phase of MMT [72]. Unfortunately, the author did not measure methadone treatment-related side effects; therefore, comparison of reported adverse events between groups and causality assessment became impossible. It is difficult for us to distinguish whether these symptoms are caused by AT because the time relationship between symptom onset and the administration of AT was not reported, and the information on dechallenge and/or rechallenge was lacking.

Comparing with auricular acupuncture, auricular acupressure is noninvasive and much easier to access. In addition to qualified AT practitioners, auricular acupressure is also widely conducted by other healthcare professionals or even unqualified persons [12]. In our analyzed studies, auricular acupressure was administered by acupuncturists, TCM practitioners, physicians, therapists, and registered nurses; however, more than half (52.4%) of the studies employing auricular acupressure failed to specify the person who performed AT, which made us unable to analyze whether there were any potentially avoidable adverse events caused by unqualified practitioners. In terms of the risk-benefit balance, either auricular acupuncture or auricular acupressure showed positive effects in managing a variety of health problems in our analyzed studies. However, for the adverse events, cases of local pain and dizziness were much less in auricular acupressure studies compared with those identified in studies on auricular acupuncture, and there were no bleeding and infection reports related to auricular acupressure. According to our findings, it seems that auricular acupressure is superior to other invasive AT approaches regarding its safety and convenience.

Adverse skin reactions, such as itchiness, redness, or allergy, were the most frequently reported adverse events in auricular acupressure, and the majority were associated with the adhesive tape used for taping acupressure seeds, while there were also some rare reports that skin irritation was caused by allergy to magnetic pellets. Despite the frequent complaints on skin irritations, these symptoms could be easily handled by changing adhesive tapes to desensitization textures or changing magnetic pellets to plant seeds. Dizziness was also reported in auricular acupressure, but the frequency was much lower than that reported in auricular acupuncture. In one case report [22], the author analyzed that dizziness might be due to the continual stimulation of the sympathetic nerve fibers wrapped around the vasa labyrinthi which could narrow blood vessels and reduce labyrinth's blood supply and, hence, lead to transient loss of orientation. This indicated that dizziness might be partially managed by reducing the frequency and intensity of acupressure.

Two case reports mentioned somnolence or drowsiness caused by auricular acupressure; the author in one study explained that it might be because of the long-term manipulation of the taped seeds inhibiting the normal functions of the autonomic nerve [23]. These kinds of symptoms were also reported in body acupuncture treatment [66, 73], and the activation of central 5-hydroxytryptamine (5-HT) pathways from the raphe nuclei may partially be involved in the onset of drowsiness after receiving acupoint stimulation, where stimulation of the raphe nuclei and the 5-HT secretion could contribute to the symptoms of fatigue, tiredness, and drowsiness [74, 75]. AT practitioners should attach importance to these reactions as somnolence or drowsiness could lead to some dangerous outcomes such as drowsiness-related motor vehicle crash. It is noteworthy that one study [44] reported 18 cases of pressure ulcers. Auricular acupressure in this study was administered by doctors and nurses who had received training in AT. Pressure ulcers were possibly caused by the long treatment duration and continual pressure. Participants in this study received weekly AT consecutively for 8 weeks; auricular implants were kept for seven days during each treatment and participants were asked to press them three times daily. No rest period was mentioned between each treatment and the continual stimulated auricle skin might have not had enough time to heal.

AT-related harms in auricular electroacupuncture and bloodletting therapy were all transient and mild. However, this review cannot ascertain the safety of these two modalities because only four studies with a limited sample size were included for analysis, and the methodological quality of adverse events reporting was unsatisfactory.

Several limitations were identified in our analyzed articles, which could affect the strength of the evidence concluded from our findings. Participant sample sizes included in this review were relatively small, and one-third of the analyzed studies failed to specify the practitioner conducting AT. Quality of adverse events reporting in included clinical trials was generally unsatisfactory, the majority did not prespecify AT-related adverse events, more than half failed to report approaches for collecting and analyzing adverse events, time relationship between the onset of symptoms and the administration of AT was not clearly described, and one-third failed to report outcomes of adverse events, all of which made it difficult to evaluate the causality of adverse events reported in clinical trials. Also, it is noted that several types of needles were utilized in studies on auricular acupuncture, and some adverse events may be associated with the variations on the length and thickness of particular AT needle, depth of insertion, and the frequency and intensity of manual pressing on the needled area. However, the information was described insufficiently in the analyzed studies, which inhibited us to judge whether there were any adverse events associated with particular types of AT equipment and procedure. Meanwhile, certain information such as the particular AT practitioner, patient's medical history, details of AT procedure and equipment, and the adverse events outcome also failed to be reported clearly in the included case reports. Moreover, there was a possibility that AT-related adverse events were underreported because during the process of the literature sorting we found a great number of papers not including adverse events as the study outcome. In addition, case reports on AT-related adverse events were rare in the published literature which made us unable to access any new or previously unrecognized harms associated with AT.

This review itself also has some limitations. Although we have made efforts to retrieve all the relevant literature, the included studies were only English and Chinese articles, and language bias may occur. Meanwhile, as there is no specific tool for the causality assessment of adverse events reported in acupuncture (including AT) studies, we employed the tool developed by the WHO-UMC in this review; however, this tool is particularly designed for adverse drug reactions and some items might not be sensitive enough for evaluating acupuncture or AT-related adverse events. Furthermore, the originally designed outcome on the frequency of AT-related adverse events was finally not calculated, as the majority of the included literature only reported the absolute number of cases complaining of adverse events.

## 5. Implications for Future Research and Practice

This study has some implications for future research and practice. Firstly, invasive AT approaches such as auricular acupuncture (manual or electric), and bloodletting therapy, should be applied by qualified practitioners. Even for the one administering noninvasive AT like auricular acupressure, receiving AT training from a qualified practitioner before application is essential because nonstandardized practice could create more unintended harms to patients. Secondly, the patients' condition needs to be well assessed before applying AT. Practitioners should identify beforehand if any clients are allergic to steel needles, adhesive tapes, magnetic pellets, or other AT materials. For weakened or elderly patients undergoing AT, semireclining position should be adopted to prevent potential harm induced by dizziness, and invasive AT should be applied with caution on patients with poor wound-healing capacity. Thirdly, the importance of patients' education should be emphasized, and the informed consent should be provided before AT. Patients need to be well informed about the potential risks associated with adverse events such as dizziness, somnolence, and drowsiness. Working at heights or driving should be avoided if patients are undergoing AT treatment. Fourthly, treatment duration and pressure intensity of AT need to be reasonably arranged. Patients should avoid putting excessive pressure on the implanted needles or taped seeds, and* de qi* sensation (a subjective feeling of numbness, pressure sensation, heaviness, soreness, or distension) could be adopted as an indicator of stopping pressure. For those receiving long-term AT treatment, a reasonable rest period between each treatment course should be provided. Moreover, for future case reports on AT-related adverse events, more details on the qualification of the practitioner, the targeted auricular acupoints, the AT equipment, the instruction on manual press, the position of the patient during AT, and the outcome of adverse events should be fully reported, and related guideline such as* the Guidelines for Case Reports of Adverse Events Related to Acupuncture* could be considered [65]. Lastly, prospective or retrospective surveys on AT-related adverse events are needed in future research to gather practitioner-reported and/or patient-reported outcomes. Future clinical trials on AT should include safety assessment as an important outcome measure, and related international guidelines such as* the CONSORT for Harms Data Recommendations* [20] should be followed to report and analyze AT-related adverse events.

## Figures and Tables

**Table 1:** Adverse events associated with auricular therapy reported in case reports.

Study and setting	Age (gender)	Reason for AT	Type of AT (method and duration)	Practitioner	Selected acupoints	AEs and severity	Outcomes	Causality^*^
Ye, 2014 [22], Chinese PLA General Hospital, Beijing, China	48 (female)	Constipation	Method: auricular acupressure using auricular plaster with vaccaria seeds Duration: not reported	Physician	*Shenmen*, kidney, liver, spleen, stomach, temple, subcortex, forehead, occiput	Event(s): dizziness Severity: not reported	Recovery after removing taped seeds	Probable/likely

Yu and Xie, 2010 [23], Teda Hospital, Tianjin, China	41 (male)	Lumbar muscle strain	Method: auricular acupressure using auricular plaster with vaccaria seeds Duration: three days per treatment, 4 treatments in total (12 days)	Physician	Heart, liver, spleen, occiput, *shenmen*, sympathetic	Event(s): somnolence Severity: not reported	Recovery after removing taped seeds	Probable/likely

Huo et al., 2000 [24], Chinese PLA 546 Hospital, Xinjiang, China	43 (male)	Dilated cardiomyopathy	Method: auricular acupressure using auricular plaster with vaccaria seeds Duration: three days per treatment, 5 treatments in total (15 days)	Not reported	Heart, liver, lung, *shenmen*, spleen, sympathetic, occiput	Event(s): somnolence Severity: not reported	Recovery after removing taped seeds	Probable/likely

Ma, 1992 [25], Ningyang TCM Hospital, Shandong, China	58 (female)	Chronic diarrhea	Method: auricular acupressure using auricular plaster with vaccaria seeds Duration: 30 minutes	Not reported	Large intestine, small intestine, *shenmen*, liver, sympathetic, spleen	Event(s): abdominal pain Severity: severe	Recovery after removing taped seeds	Possible

AT: auricular therapy, AE: adverse event, PLA: People's Liberation Army, and TCM: traditional Chinese medicine.

^*^The WHO-Uppsala Monitoring Centre (UMC) System for Standardized Case Causality Assessment: certain—a plausible time relationship that adverse events clearly occurred after receiving AT and disappeared after withdrawal, and these events could not be explained by other health problems or interventions; probable/likely—a reasonable time relationship that the onset of symptoms was most likely related to AT and that was unlikely attributed to other health problems or interventions; possible—a reasonable time relationship that the onset of symptoms was most likely related to AT but that could also be explained by other health problems or interventions, and the information at withdrawal was lacking or unclear; unlikely—there was an improbable time relationship between AT and the adverse event; conditional/unclassified—event occurred but more data were essential for a proper causality assessment; unassessable/unclassifiable—an adverse event was suggested by a report but cannot be judged due to insufficient or contradictory information.

**Table 2:** Adverse events associated with auricular therapy reported in clinical trials.

Study	Reason for AT and practitioner	Intervention (details of AT) and control	Selected acupoints	AEs (number of Cases) of AT and outcome	Quality of AEs Reporting^△ ^
Studies on auricular acupuncture					
Prisco et al., 2013 [26] RCT (*N* = 35) Department of Veterans Affairs Medical Center, Washington DC, USA	R: PTSD-related insomnia P: physician	Intervention (true AT): (i) Method: auricular acupuncture with spring handle needles (0.16 × 15 mm) (ii) Duration: two treatments weekly (45 minutes per treatment) for 8 weeks Control 1 (sham AT): auricular acupuncture at nonacupuncture points Control 2: waiting-list control	True AT: *shenmen*, liver, kidney, sympathetic, hippocampus Sham AT: nonacupuncture points located on the helix of the ear	Uncomfortable at the needling site (*n* = 1) Outcome: withdrawal	(1) Not reported (2) Not reported (3) Not reported (4) Not reported (5) Not reported (6) Adequate (7) Not reported

Lua and Talib, 2013 [27] RCT (*N* = 97) Three Methadone Maintenance Treatment (MMT) Centers, Terengganu, Malaysia	R: drug dependence P: acupuncturist	Intervention (MMT + AT): (i) Method: auricular acupuncture (needles: 0.25 × 12.55 mm) (ii) Duration: three treatments weekly (30 minutes per treatment) for 8 weeks Control: MMT only	*Shenmen*, sympathetic, kidney, liver, lung	Light headache (*n* = 17) Slight bleeding (*n* = 14) Tingling sensations (*n* = 19) Pain (*n* = 17) and nausea (*n* = 19) Drowsiness (*n* = 11) and dizziness (*n* = 19) Dry mouth (*n* = 15) and slight fever (*n* = 19) Outcome: not reported	(1) Adequate (2) Adequate (3) Not reported (4) Adequate (5) Adequate (6) Inadequate (7) Adequate

Hunter et al., 2012 [28] RCT (*N* = 52) Primary Care & A University Population, Northern Ireland, UK	R: chronic low back pain P: physiotherapist	Intervention (exercise + AT): (i) Method: auricular acupuncture with Seirin Pyonex ear needles (1.80 × 0.26 mm) (ii) Duration: one treatment (48 hours) weekly for 6 weeks Control: exercise only	*Shenmen*, lumbar spine, cushion	Pain and redness (not reported) Minor bleeding (not reported) Swelling around the needling site (*n* = 1)^#^ Outcome: (i) NR (for pain, redness, and bleeding) (ii) Recovery (for swelling)	(1) Adequate (2) Not reported (3) Not reported (4) Not reported (5) Not reported (6) Inadequate (7) Partially adequate

Michalek-Sauberer et al., 2012 [29] RCT (*N* = 182) Outpatient Clinic, Medical University of Vienna, Vienna, Austria	R: state anxiety before dental treatment P: investigator with a diploma of acupuncture	Intervention (true AT): (i) Method: auricular acupuncture (needles: 0.2 × 15 mm) (ii) Duration: twenty minutes until the start of dental treatment Control 1 (sham AT): auricular acupuncture at nonspecific points Control 2: no intervention	True AT: relaxation, tranquilizer, master cerebral Sham AT: tonsil, finger, shoulder	Warmth or a strange feeling at the treated ear or dizziness (*n* = 26) Outcome: not reported	(1) Not reported (2) Not reported (3) Not reported (4) Not reported (5) Not reported (6) Not reported (7) Not reported

Lien et al., 2012 [30] RCT (*N* = 90) Taipei City Hospital, Taiwan	R: obesity P: acupuncturist	Intervention (true AT): (i) Method: auricular acupuncture (needles: 2 mm tip and 10 mm annular-shaped body) (ii) Duration: three treatments weekly for 4 weeks Control 1: auricular stimulation using magnetic pellets Control 2 (sham AT): needles with the tips removed	*Shenmen*, stomach, hunger, endocrine	Dizziness (*n* = 1) Outcome: withdrawal	(1) Adequate (2) Partially adequate (3) Not reported (4) Not reported (5) Not reported (6) Adequate (7) Not reported

Hsu et al., 2009 [31] RCT (*N* = 60) Taipei Hospital, Taiwan	R: obesity P: acupuncturist	Intervention (true AT): (i) Method: auricular acupuncture (needles: 2 mm tip and 10 mm annular-shaped body) (ii) Duration: two treatments weekly (3 days per treatment) for 6 weeks Control (sham AT): needles with the tips removed	*Shenmen*, stomach, hunger, endocrine	Minor inflammation at the needling site (*n* = 1) Mild tenderness at the needling site (*n* = 9) Outcome: not reported	(1) Adequate (2) Not reported (3) Not reported (4) Not reported (5) Not reported (6) Adequate (7) Not reported

Wang et al., 2009 [32] RCT (*N* = 159) Yale-New Haven Hospital, New Haven, USA	R: pregnant women with low back pain and posterior pelvic pain P: acupuncturist	Intervention (true AT): (i) Method: auricular acupuncture with Seirin Pyonex ear needles (ii) Duration: one week Control 1 (sham AT): auricular acupuncture at nonspecific points Control 2: no treatment	True AT: *shenmen*, kidney, analgesia Sham AT: shoulder, wrist, extra-auricular point	Transient ear tenderness (*n* = 4) Outcome: resolved spontaneously	(1) Not reported (2) Not reported (3) Adequate (4) Adequate (5) Not reported (6) Not reported (7) Partially adequate

Harding et al., 2008 [33] Non-RCT (*N* = 60) James Cook University Hospital, Middlesbrough, UK	R: hot flushes in prostate cancer patients with LHRH agonist treatment P: not reported	Intervention (AT): (i) Method: auricular acupuncture with 0.20 g sterile single-use needles (ii) Duration: weekly treatment (40 minutes) for 10 weeks Control: not applicable	*Shenmen*, liver, lung, autonomic, kidney	Transient exacerbation of vasomotor symptoms (*n* = 2) Outcome: resolved spontaneously (lasted only seconds)	(1) Adequate (2) Not reported (3) Not reported (4) Not reported (5) Not reported (6) Adequate (7) Partially adequate

Courbasson et al., 2007 [34] Non-RCT (*N* = 305) The Jean Tweed Centre, Toronto, Ontario, Canada	R: women with concurrent substance use problems and anxiety and depressive symptoms P: acupuncturist	Intervention (AT + usual treatment): (i) Method: auricular acupuncture (details not described) (ii) Duration: three treatments weekly (45 minutes per treatment) (length of treatment not mentioned) Control: usual treatment	Not reported	Experiencing pain from needles (not reported) Outcome: not reported	(1) Not reported (2) Inadequate (3) Not reported (4) Partially adequate (5) Not reported (6) Inadequate (7) Not reported

Wu et al., 2007 [35] RCT (*N* = 131) Smoking Cessation Clinics, Taipei Veterans General Hospital, Taiwan	R: smoking cessation P: acupuncturist	Intervention (true AT): (i) Method: auricular acupuncture with 36 gauge 0.5 inch ear-piercing needles (ii) Duration: weekly treatment for 8 weeks Control (sham AT): auricular acupuncture at nonspecific points	True AT: *shenmen*, lung, mouth, sympathetic Sham AT: eye, elbow, shoulder, knee	Hematoma (*n* = 1) Feeling of residual needling (*n* = 24) Tenderness sensation (*n* = 50) Minor bleeding (*n* = 3) Minor infection (*n* = 1) Nausea (*n* = 2) and dizziness (*n* = 4) Outcome: gradually declined	(1) Adequate (2) Not reported (3) Adequate (4) Adequate (5) Adequate (6) Inadequate (7) Adequate

Usichenko et al., 2007 [36] RCT (*N* = 120) Ambulatory Orthopedic Surgery Center of the Ernst Moritz Arndt University, Greifswald, Germany	R: postoperative pain P: acupuncturist	Intervention (true AT + analgesia): (i) Method: auricular acupuncture with indwelling steel needles (0.22 × 1.5 mm) (ii) Duration: inserted before surgery and kept until the following morning Control (sham AT + analgesia): auricular acupuncture at nonacupuncture points	True AT: *shenmen*, lung, knee joint Sham AT: nonacupuncture points located on the helix ipsilateral to the site of surgery	Dizziness and nausea (*n* = 1) Pain at insertion and sleep disturbance (*n* = 1) Outcome: (i) Disappeared after withdrawal of the needles (for dizziness and nausea) (ii) Not reported (for pain and sleep disturbance)	(1) Not reported (2) Not reported (3) Not reported (4) Not reported (5) Partially adequate (6) Partially adequate (7) Adequate

Kunz et al., 2007 [37] RCT (*N* = 109) Clinic of Psychiatry and Psychotherapy Bethel, Bielefeld, Germany	R: alcohol withdrawal P: psychiatrists or mental-health nurses	Intervention (AT + usual treatment): (i) Method: auricular acupuncture with stainless-steel acupuncture needles (0.2 × 0.15 mm) (ii) Duration: daily treatment (45 minutes) for 5 consecutive days Control: aromatherapy + usual treatment	*Shenmen*, sympathetic, kidney, liver, lung	Pain and mild bleeding (*n* = 6) Outcome: not reported	(1) Not reported (2) Not reported (3) Not reported (4) Adequate (5) Partially adequate (6) Inadequate (7) Adequate

Usichenko et al., 2005 [38] RCT (*N* = 61) Department of Anesthesiology and Orthopedic Surgery, University of Greifswald, Germany	R: postoperative pain P: acupuncturist	Intervention (true AT + analgesia): (i) Method: auricular acupuncture with permanent press steel needles (0.22 × 1.5 mm) (ii) Duration: inserted the evening before surgery and kept for 3 days after surgery Control (sham AT + analgesia): auricular acupuncture at nonacupuncture points	True AT: *shenmen*, lung, thalamus, hip joint Sham AT: nonacupuncture points on the auricular helix	Pain at the needling site (*n* = 3) Minor bleeding at the needling site (*n* = 2) Headache (*n* = 1) Hip pain after needle withdrawal (*n* = 2) Outcome: (i) One withdrew, not reported for another 2 (for pain) (ii) Recovery after treatment (for bleeding) (iii) Recovery after surgery (for headache) (iv) Not reported (for hip pain)	(1) Not reported (2) Not reported (3) Not reported (4) Not reported (5) Inadequate (6) Adequate (7) Inadequate

Berman et al., 2004 [39] RCT (*N* = 158) Two Medium-Security Institutions (Jails), Sweden	R: drug use problem, psychological symptoms, and physical discomfort in prison inmates P: acupuncturist	Intervention (true AT): (i) Method: auricular acupuncture with stainless-steel disposable needles (0.22 × 0.13 mm) (ii) Duration: a total of 14 treatments (40 minutes per treatment) for 4 weeks Control (sham AT): auricular acupuncture at nonspecific points	True AT: *shenmen*, lung, liver, kidney, sympathetic Sham AT: nonspecific points on the auricular helix	Pain at insertion (*n* = 44) Outcome: forty-two dropped out and 2 completed treatment even though they found it painful	(1) Adequate (2) Adequate (3) Adequate (4) Adequate (5) Partially adequate (6) Inadequate (7) Adequate

Bier et al., 2002 [40] RCT (*N* = 141) Arizona, USA	R: smoking cessation and cigarette consumption P: acupuncturist	Intervention 1 (true acupuncture + education): (i) Method: auricular acupuncture with 36 gauge 0.5 inch needles (ii) Duration: five treatments weekly (30 minutes per treatment) for 4 weeks Intervention 2 (true acupuncture) Control (sham acupuncture + education): auricular acupuncture at nonacupuncture points	True AT: *shenmen*, lung, liver, kidney, sympathetic Sham AT: nonacupuncture points located within 5 mm of the true points	Infrequent minor bleeding upon needle removal (not reported) Outcome: not reported	(1) Not reported (2) Not reported (3) Not reported (4) Adequate (5) Not reported (6) Inadequate (7) Not reported

Gurevich et al., 1996 [41] Non-RCT (*N* = 77) North Shore University Hospital at Glen Cove, USA	R: substance-abuse problem P: psychiatrist and nurses	Intervention (receiving AT for 5 or more times): (i) Method: auricular acupuncture with sterile disposable needles (ii) Duration: daily treatment (20–40 minutes) Control: receiving AT for 4 or fewer times	*Shenmen*, lung, liver, kidney, sympathetic	Minor local bleeding (not reported) Local pain (not reported) Outcome: (i) Treatment was not required (for bleeding) (ii) Stopped AT temporarily or had less frequent treatments (for local pain)	(1) Partially adequate (2) Inadequate (3) Partially adequate (4) Not reported (5) Not reported (6) Partially adequate (7) Not reported

Washburn et al., 1993 [42] RCT (*N* = 100) The Bayview-Hunters Point Foundation, San Francisco, USA	R: heroin addiction P: acupuncturist	Intervention (true AT + support service): (i) Method: auricular acupuncture with single-use disposable needles (ii) Duration: twenty-one days (20–45 minutes per treatment) Control (sham AT + support service): auricular acupuncture at nonacupuncture points	True AT: *shenmen*, lung, kidney, sympathetic Sham AT: nonacupuncture points located close to the true points	Slight bleeding at insertion (not reported) Mild nausea and dizziness (not reported) Outcome: relief when the needles were removed	(1) Not reported (2) Inadequate (3) Partially adequate (4) Not reported (5) Not reported (6) Inadequate (7) Not reported

Zhang and Fan, 1986 [43] Non-RCT (*N* = 179) TCM Academy of Shanxi Province, China	R: cholecystolithiasis P: TCM practitioner	Intervention (AT): (i) Method: auricular acupuncture for main points and auricular acupressure for adjunct points (details were not described) (ii) Duration: not reported Control: not applicable	Liver, gallbladder, stomach, duodenum, *shenmen*, sympathetic, lung	Dizziness, upper limb numbness, and minor nausea (not reported) Outcome: disappeared after removing stimulation on acupoint “sympathetic”	(1) Not reported (2) Not reported (3) Not reported (4) Not reported (5) Not reported (6) Inadequate (7) Not reported

Studies on auricular acupressure					
Vas et al., 2014 [44] RCT (*N* = 265) Ten Primary Healthcare Centres, Seville, Spain	R: chronic nonspecific spinal pain P: doctors and nurses with AT training	Intervention (true AT): (i) Method: auricular acupressure using auricular plaster with vaccaria seeds (ii) Duration: weekly treatment (seeds kept for 7 days) for 8 weeks Control (placebo AT): auricular acupressure using auricular plaster with inactive black plastic discs	Main acupoints: *shenmen*, thalamus Adjunct acupoints: not reported	Pressure ulcers in the pinna (*n* = 18) Worsened symptoms (*n* = 8) Outcome: (i) Healed within 10 days of removal (for pressure ulcers) (ii) Not reported (for worsened symptoms)	(1) Adequate (2) Adequate (3) Not reported (4) Adequate (5) Inadequate (6) Inadequate (7) Partially adequate

Li et al., 2014 [45] RCT (*N* = 99) Elderly Residential Care Home, Hong Kong	R: constipation P: not reported	Intervention (true AT): (i) Method: auricular acupressure using auricular plaster with magnetic pellets (ii) Duration: ten days Control 1 (placebo AT): auricular acupressure using auricular plaster with vaccaria seeds Control 2 (usual care): auricular acupressure using auricular plaster only	Large intestine, rectum, *San Jiao*, spleen, lung, sympathetic, subcortex	Minor local itchiness (*n* = 27) Minor dizziness (*n* = 2) Outcome: (i) Spontaneously subsided after AT (25) and 2 withdrew (for itchiness) (ii) Spontaneously subsided after AT (1) and 1 withdrew (for dizziness)	(1) Partially adequate (2) Partially adequate (3) Adequate (4) Not reported (5) Inadequate (6) Adequate (7) Partially adequate

Zhang et al., 2013 [46] RCT (*N* = 43) RMIT University, Australia	R: smoking cessation P: acupuncturist	Intervention (true AT): (i) Method: auricular acupressure with stainless-steel press-pellet tapes (ii) Duration: weekly treatment for 8 weeks Control (sham AT): auricular acupressure at nonspecific points	True AT: *shenmen*, lung, mouth, extra, liver Sham AT: helix 2, shoulder, clavicle, occiput, tooth	Mild to moderate local discomfort (*n* = 5) Slight headache and dizziness (*n* = 1) Outcome: (i) All AEs were resolved without any medical intervention (for both) (ii) One subject withdrew (for ear discomfort)	(1) Adequate (2) Adequate (3) Not reported (4) Adequate (5) Inadequate (6) Adequate (7) Partially adequate

Kong, 2012 [47] RCT (*N* = 60) Foshan Hospital of TCM, Foshan, China	R: postoperative pain P: not reported	Intervention (AT + intravenous analgesia): (i) Method: auricular acupressure using auricular plaster with vaccaria seeds (ii) Duration: one treatment (5 hours after surgery), seeds kept for 3 days Control: intravenous analgesia only	*Shenmen*, liver, kidney, heel, lesser occipital nerve, great auricular nerve	Mild skin irritation (*n* = 2) Outcome: no obvious discomfort and AT continued	(1) Adequate (2) Partially adequate (3) Not reported (4) Adequate (5) Partially adequate (6) Inadequate (7) Inadequate

Yeh et al., 2012 [48] RCT (*N* = 10) A Large Children's Hospital, Taiwan	R: chemotherapy-induced nausea and vomiting P: therapist	Intervention (true AT + standard care): (i) Method: auricular acupressure using auricular plaster with plant seeds (ii) Duration: one treatment, seeds kept for 7 days Control (sham AT + standard care): auricular acupressure at nonspecific points	True AT: *Shenmen*, sympathetic, cardia, stomach, digestive subcortex Sham AT: external knee point, vision, shoulder joint, eye	Local itchiness (*n* = 3) Outcome: continued to complete the study	(1) Not reported (2) Not reported (3) Not reported (4) Not reported (5) Not reported (6) Inadequate (7) Not reported

Li et al., 2012 [49] RCT (*N* = 39) Elderly Residential Care Home, Hong Kong	R: constipation P: not reported	Intervention (true AT): (i) Method: auricular acupressure using auricular plaster with magnetic pellets (ii) Duration: three weeks Control (placebo AT): auricular acupressure using auricular plaster with vaccaria seeds	Large intestine, rectum, *San Jiao*, spleen, lung, sympathetic, subcortex	Mild, tolerable, and short-term itchiness of the ears (*n* = 7) Outcomes: not reported	(1) Not reported (2) Not reported (3) Not reported (4) Partially adequate (5) Not reported (6) Inadequate (7) Not reported

Jin et al., 2012 [50] RCT (*N* = 80) Cangnan TCM Hospital of Zhejiang Province, Cangnan, China	R: severe insomnia P: TCM practitioner	Intervention (AT): (i) Method: auricular acupressure using auricular plaster with magnetic pellets (ii) Duration: one treatment, seeds kept for 7 days Control: standardized medication	Main acupoints: *shenmen*, occiput, subcortex, sympathetic Adjunct acupoints: (based on TCM syndrome) heart, spleen, kidney, liver, stomach	Local redness at the taped site (*n* = 2) Outcome: recovery after treatment	(1) Inadequate (2) Not reported (3) Not reported (4) Adequate (5) Not reported (6) Inadequate (7) Not reported

Kung et al., 2011 [51] Non-RCT (*N* = 45) Taipei Veterans General Hospital, Taiwan	R: women with postmenopausal insomnia P: acupuncturist	Intervention (AT): (i) Method: auricular acupressure using auricular plaster with magnetic pellets (ii) Duration: daily treatment (every night before sleep) for 4 weeks Control: not applicable	*Shenmen*, kidney, heart, brainstem, subcortex	Sensation of auricular tenderness (*n* = 2) Outcome: not reported	(1) Not reported (2) Not reported (3) Not reported (4) Not reported (5) Not reported (6) Inadequate (7) Not reported

Xia et al., 2011 [52] RCT (*N* = 60) Baoan Hospital Affiliated to South Medical University, Shenzhen, China	R: low back pain caused by lumbar strain P: not reported	Intervention (AT + Chinese medicine plaster): (i) Method: auricular acupressure using auricular plaster with vaccaria seeds (ii) Duration: two treatments weekly for 2 weeks Control: Chinese medicine plaster only	Ashi point, kidney, liver, lumbosacral vertebrae, *shenmen*, subcortex	Obvious pain at the taped site when receiving AT for the first time (*n* = 5) Outcome: completed the study by reducing pressing frequency and intensity	(1) Adequate (2) Not reported (3) Partially adequate (4) Partially adequate (5) Not reported (6) Inadequate (7) Not reported

Xue et al., 2011 [53] RCT (*N* = 63) Two Metropolitan RMIT Campus in Melbourne, Australia	R: persistent allergic rhinitis P: acupuncturist	Intervention (true AT): (i) Method: auricular acupressure using auricular plaster with stainless-steel pellets (ii) Duration: eight weeks Control (sham AT): auricular acupressure at nonspecific points	True AT: *shenmen*, internal nose, lung, wind stream Sham AT: adrenal gland, helix 2, shoulder, clavicle, occiput, teeth	Mild to moderate local and short-term discomfort (*n* = 30) Sore ear (*n* = 9) Ear itch (*n* = 7) Outcome: well tolerated	(1) Not reported (2) Not reported (3) Not reported (4) Partially adequate (5) Not reported (6) Inadequate (7) Inadequate

Ji et al., 2010 [54] RCT (*N* = 73) Shuguang Hospital Affiliated to Shanghai University of TCM, Shanghai, China	R: functional constipation P: nurse	Intervention (AT + usual care): (i) Method: auricular acupressure using auricular plaster with Liu Shen Wan (for excess syndrome) or magnetic pellets (for deficiency syndrome) (ii) Duration: one month Control: usual care	Main acupoints: large intestine, small intestine, rectum Adjunct acupoints: Lung, *San Jiao*, stomach (for excess syndrome), spleen, kidney, endocrine (for deficiency syndrome)	Mild redness and skin breakdown at the taped site (*n* = 1) Outcome: recovery two days later after using entoiodine	(1) Adequate (2) Not reported (3) Not reported (4) Adequate (5) Not reported (6) Inadequate (7) Not reported

Wing et al., 2010 [55] RCT (*N* = 70) Outpatient Clinics and The Community, Hong Kong	R: smoking cessation P: not reported	Intervention (true AT + hand acupressure): (i) Method: auricular acupressure using auricular plaster with hard beads (ii) Duration: three weeks Control (sham AT + sham hand acupressure): auricular acupressure at nonmeridian points	True AT: *shenmen*, lung, month, brain Nonmeridian points: away from those selected for the treatment group	Skin irritation (allergy) at the site of the adhesive tapes (*n* = 3) Outcome: withdrawal	(1) Adequate (2) Partially adequate (3) Not reported (4) Not reported (5) Not reported (6) Adequate (7) Partially adequate

Sun, 2010 [56] RCT (*N* = 173) Department of TCM, Xuzhou First Hospital of Jiangsu Province, Xuzhou, China	R: insomnia P: not reported	Intervention (AT + psychological support): (i) Method: auricular acupressure using auricular plaster with vaccaria seeds (ii) Duration: twenty days Control: AT only	Main acupoints: *shenmen*, sympathetic, endocrine, heart, subcortex Adjunct acupoints: liver, stomach, spleen, kidney, pancreas and gallbladder, heart of dorsal surface	Skin irritation (allergy) at the site of the adhesive tapes (*n* = 5) Outcome: changed to desensitization tapes and treatment continued	(1) Not reported (2) Not reported (3) Not reported (4) Not reported (5) Not reported (6) Inadequate (7) Not reported

Peng, 2009 [57] Non-RCT (*N* = 30) Hanguang Hospital of Handan City, Handan, China	R: neurasthenia P: not reported	Intervention (AT): (i) Method: auricular acupressure using auricular plaster with magnetic pellets (ii) Duration: three days per treatment, 20 treatments in total (60 days) Control: not applicable	Main acupoints: *shenmen*, subcortex, endocrine, anterior ear lobe Adjunct acupoints: (based on TCM syndrome) liver, gallbladder, *San Jiao*, stomach, lung, heart	Skin allergy and itchiness of the ear (not reported) Outcome: symptom disappeared after changing magnetic pellets to vaccaria seeds	(1) Not reported (2) Not reported (3) Not reported (4) Not reported (5) Not reported (6) Inadequate (7) Not reported

Chen et al., 2009 [58] RCT (*N* = 180) TCM Hospital of Hainan Province, Haikou, China	R: vascular dementia P: TCM practitioner	Intervention (AT): (i) Method: auricular acupressure using auricular plaster with vaccaria seeds (ii) Duration: daily treatment for 12 weeks Control: standardized medication	*Shenmen*, brain, kidney, occiput	Severe skin allergy and itchiness at the taped site (*n* = 2) Outcome: withdrawal	(1) Adequate (2) Partially adequate (3) Not reported (4) Inadequate (5) Not reported (6) Adequate (7) Not reported

Wang et al., 2007 [59] RCT (*N* = 198) People's Hospital of Baoshan City, Baoshan, China	R: myopia P: not reported	Intervention (AT): (i) Method: auricular acupressure using auricular plaster with vaccaria seeds (ii) Duration: four weeks for one treatment Control: standardized medication	Apex of ear, kidney, liver, eye, eye 2, spleen,	Skin allergy and local redness at the taped site (*n* = 2) Outcome: not reported	(1) Adequate (2) Not reported (3) Not reported (4) Not reported (5) Not reported (6) Inadequate (7) Not reported

Ding et al., 2006 [60] RCT (*N* = 200) TCM Hospital of Hebei Province, Shijiazhuang, China	R: motion sickness P: acupuncturist	Intervention (AT + Neiguan acupressure): (i) Method: auricular acupressure using auricular plaster with magnetic pellets (ii) Duration: not reported Control: standardized medication	Stomach, occiput, *shenmen*, sympathetic	Itchiness at the taped site (*n* = 2) Outcome: not reported	(1) Adequate (2) Not reported (3) Not reported (4) Not reported (5) Not reported (6) Inadequate (7) Not reported

Studies on auricular electroacupuncture					
Schukro et al., 2013 [61] RCT (*N* = 56) Department of Special Anesthesia and Pain Management at the Medical University of Vienna, Vienna, Austria	R: obesity in female patients P: not reported	Intervention (AT + diet based on TCM): (i) Method: auricular acupuncture with electrical stimulation using P-stim electroacupuncture device (needle: 27 gauge, 3 mm length) (ii) Duration: 4-day treatment per week for 6 weeks Control (placebo AT + diet based on TCM): auricular acupuncture with a P-stim dummy	Hunger, stomach, colon	mild skin irritations behind the ear caused by the adhesive patch of the P-stim/placebo device (*n* = 8) Outcome: resolved immediately after the end of application	(1) Adequate (2) Not reported (3) Not reported (4) Not reported (5) Not reported (6) Inadequate (7) Not reported

Fritz et al., 2013 [62] RCT (*N* = 125) St. Louis Veterans Affairs Medical Center, USA	R: smoking cessation P: registered nurse	Intervention (AT): (i) Method: auricular acupuncture with the Stim Flex 400A Transcutaneous Electrical Nerve Stimulation Unit (active, 80 Hz) (ii) Duration: weekly treatment (20 minutes per treatment) for 5 consecutive weeks Control (sham AT): auricular acupuncture with the Stim Flex 400A Transcutaneous Electrical Nerve Stimulation Unit (inactive, 0 Hz)	Lung, *shenmen*, nicotine, point zero, palate	Auricle discomfort without redness or swelling (*n* = 1) Outcome: not reported	(1) Adequate (2) Not reported (3) Not reported (4) Partially adequate (5) Not reported (6) Partially adequate (7) Partially adequate

Bernateck et al., 2008 [63] RCT (*N* = 44) Outpatient Clinic of the Department of Rheumatology, Hannover Medical School, Hannover, Germany	R: rheumatoid arthritis P: doctor with profound acupuncture experience	Intervention (AT): (i) Method: auricular acupuncture with electrical stimulation using P-stim device (needle: 27 gauge, 3 mm length) (ii) Duration: weekly treatment (48 hours per treatment) for 6 weeks Control: autogenic training	S*henmen*, cushion, an individual point depending on the main pain spots	Pain and discomfort at the needling site (*n* = 1) Outcome: not reported	(1) Not reported (2) Not reported (3) Not reported (4) Not reported (5) Not reported (6) Inadequate (7) Not reported

Studies on auricular bloodletting therapy					
Yuan and Qiao, 1998 [64] Non-RCT (*N* = 170) Chinese PLA 34260 Hospital, Xiangfan, China	R: acute tonsillitis P: not reported	Intervention (AT): (i) Method: auricular bloodletting therapy with three-edged needle (ii) Duration: not applicable Control: not applicable	Helix 6 (Ashi point)	Minor infection at the needling site (*n* = 2) Outcome: not reported	(1) Not reported (2) Not reported (3) Not reported (4) Not reported (5) Not reported (6) Inadequate (7) Not reported

AT: auricular therapy, AE: adverse event, RCT: randomized controlled trial, R: reason for AT, PTSD: posttraumatic stress disorder, P: practitioner, LHRH: luteinizing hormone releasing hormone, and TCM: traditional Chinese medicine.

^△  ^The CONSORT Recommendation for AEs: (1) report of data on harms in the title or abstract, (2) report of AT-related harms in the introduction section, (3) prespecification of potential adverse events of AT (clinical and/or laboratory), (4) specification of approach for collecting harms-related information, (5) description of plans for presenting and analyzing adverse events of AT, (6) description of participant withdrawals due to adverse events of AT, and (7) report of the particular denominators for analyses on AT-related harms. Quality grades for each item: adequate—item was properly described in detail in the article or in the study protocol; partially adequate—item was properly described but only in a brief format; inadequate—item failed to be properly described; not reported—item was not described.

^#^Happened in one participant who did not disclose a history of rheumatoid arthritis (one of the exclusion criteria for that study).

**Table 3:** Selected searching strategies for the systematic review.

ID	Searching strategies	Records
PubMed		
#1	“auriculotherapy”[MeSH Terms] OR “acupuncture, ear”[MeSH Terms]	**264**
#2	((((((((((((((((((auriculotherap^*^[Title/Abstract]) OR (acupunctur^*^[Title/Abstract] AND ear^*^[Title/Abstract])) OR (acupunctur^*^[Title/Abstract] AND auricu^*^[Title/Abstract])) OR (acupressur^*^[Title/Abstract] AND ear^*^[Title/Abstract])) OR (acupressur^*^[Title/Abstract] AND auricu^*^[Title/Abstract])) OR (auricu^*^[Title/Abstract] AND poin^*^[Title/Abstract])) OR (ear[Title/Abstract] AND poin^*^[Title/Abstract])) OR (auricu^*^[Title/Abstract] AND acupoin^*^[Title/Abstract])) OR (ear[Title/Abstract] AND acupoin^*^[Title/Abstract])) OR (auricu^*^[Title/Abstract] AND plaster^*^[Title/Abstract])) OR (massag^*^[Title/Abstract] AND ear^*^[Title/Abstract])) OR (ear[Title/Abstract] AND plaster^*^[Title/Abstract])) OR (massag^*^[Title/Abstract] AND auricu^*^[Title/Abstract])) OR (magne^*^[Title/Abstract] AND ear^*^[Title/Abstract])) OR (magne^*^[Title/Abstract] AND auricu^*^[Title/Abstract])) OR otopoin^*^[Title/Abstract]) OR vaccaria^*^[Title/Abstract]) OR erxue[Title/Abstract]	**35299**
#3	#1 OR #2	**35338**
#4	((((((“adverse event^*^”[Title/Abstract]) OR “adverse effect^*^”[Title/Abstract]) OR “adverse reaction^*^”[Title/Abstract]) OR “side effect^*^”[Title/Abstract]) OR “complication^*^”[Title/Abstract]) OR “safe^*^”[Title/Abstract]) OR “risk^*^”[Title/Abstract]	**1608414**
#5	#3 AND #4	**4284**

EMBase		
#1	auriculotherap^*^:ab,ti OR (ear NEAR/3 acupunctur^*^):ab,ti OR (auricu^*^ NEAR/3 acupunctur^*^):ab,ti OR (ear NEAR/3 acupressur^*^):ab,ti OR (auricu^*^NEAR/3 acupressur^*^):ab,ti OR (auricu^*^ NEAR/3 poin^*^):ab,ti OR ‘auricular plaster':ab,ti OR (ear NEAR/3 plaster^*^):ab,ti OR (ear NEAR/3 poin^*^):ab,ti OR (auricu^*^ NEAR/3 acupoint^*^):ab,ti OR (ear NEAR/3 acupoint^*^):ab,ti OR otopoin^*^:ab,ti OR (vaccaria^*^ NEAR/15 ear^*^):ab,ti OR (vaccaria^*^ NEAR/15 auricu^*^):ab,ti OR (massag^*^ NEAR/3 auricu^*^):ab,ti OR (massag^*^ NEAR/3 ear^*^):ab,ti OR (cowherb NEAR/15 ear^*^):ab,ti OR (cowherb NEAR/15 auricu^*^):ab,ti OR (magne^*^ NEAR/15 ear^*^):ab,ti OR (magne^*^ NEAR/15 auricu^*^):ab,ti OR erxue^*^:ab,ti	**9599**
#2	(adverse NEAR/3 event^*^):ab,ti OR (adverse NEAR/3 effect^*^):ab,ti OR (adverse NEAR/3 reaction^*^):ab,ti OR (side NEAR/3 effect^*^):ab,ti OR complication^*^:ab,ti OR safe^*^:ab,ti OR risk^*^:ab,ti	**3092250**
#3	#1 AND #2	**1688**

CENTRAL		
#1	MeSH descriptor: [Auriculotherapy] explode all trees	**126**
#2	MeSH descriptor: [Acupuncture, Ear] explode all trees	**120**
#3	auriculotherap^*^ or (ear near/3 acupunctur^*^) or (auricu^*^ near/3 acupunctur^*^) or (ear near/3 acupressur^*^) or (auricu^*^ near/3 acupressur^*^) or (auricu^*^ near/3 poin^*^) or (ear near/3 poin^*^) or (ear near/3 plaster^*^) or (auricu^*^ near/3 plaster^*^) or (auricu^*^ near/3 acupoint^*^) or (ear near/3 acupoint^*^) or otopoint^*^ or (vaccaria^*^ near/15 ear) or (vaccaria^*^ near/15 auricu^*^) or (cowherb near/15 ear^*^) or (cowherb near/15 auricu^*^) or (magne^*^ near/15 ear^*^) or (magne^*^ near/15 auricu^*^) or (massag^*^ near/3 ear^*^) or (massag^*^ near/3 auricu^*^) or erxue^*^:ti,ab,kw (Word variations have been searched)	**864**
#4	#1 OR #2 OR #3	**864**
#5	complication^*^ or (adverse near/3 event^*^) or (adverse near/3 effect^*^) or (adverse near/3 reaction^*^) or (side near/3 effect^*^) or safe^*^ or risk^*^: ti,ab,kw (Word variations have been searched)	**240648**
#6	#4 AND #5	**182**
#7	#6 in Trials	**158**
